# Molecular characterization and genetic diversity of four undescribed novel oleaginous *Mortierella alpina *strains from Libya

**DOI:** 10.12688/f1000research.70644.1

**Published:** 2021-09-07

**Authors:** Fuzia Elfituri Muftah Eltariki, Kartikeya Tiwari, Mohammed Abdelfatah Alhoot

**Affiliations:** 1 Microbiology and Post Graduation Center, Management and Science University, Shah Alam, Selangor, 40100, Malaysia

**Keywords:** Single cell oil, Mortierella alpina, Potato dextrose agar, Sporangiospore, Mortierellales

## Abstract

**Background:** A large number of undiscovered fungal species still exist on earth, which can be useful for bioprospecting, particularly for single cell oil (SCO) production. *Mortierella* is one of the significant genera in this field and contains about hundred species. Moreover, *M. alpina *is the main single cell oil producer at commercial scale under this genus.

**Methods:** Soil samples from four unique locations of North-East Libya were collected for the isolation of oleaginous *Mortierella* *alpina* strains by a serial dilution method. Morphological identification was carried out using light microscopy (Olympus, Japan) and genetic diversity of the isolated *Mortierella alpina* strains was assessed using conserved internal transcribed spacer (ITS) gene sequences available on the NCBI GenBank database for the confirmation of novelty. The nucleotide sequences reported in this study have been deposited at GenBank (accession no. MZ298831:MZ298835). The MultAlin program was used to align the sequences of closely related strains. The DNA sequences were analyzed for phylogenetic relationships by molecular evolutionary genetic analysis using MEGA X software consisting of Clustal_X v.2.1 for multiple sequence alignment. The neighbour-joining tree was constructed using the Kimura 2-parameter substitution model.

**Results:** The present research study confirms four oleaginous fungal isolates from Libyan soil. These isolates (barcoded as MSU-101, MSU-201, MSU-401 and MSU-501) were discovered and reported for the first time from diverse soil samples of district Aljabal Al-Akhdar in North-East Libya and fall in the class: *Zygomycetes*; order: *Mortierellales*.

**Conclusions:** Four oleaginous fungal isolates barcoded as MSU-101, MSU-201, MSU-401 and MSU-501 were identified and confirmed by morphological and molecular analysis. These fungal isolates showed highest similarity with *Mortierella alpina* species and can be potentialistic single cell oil producers. Thus, the present research study provides insight to the unseen fungal diversity and contributes to more comprehensive *Mortierella alpina* reference collections worldwide.

## Introduction

Edible oils produced by oleaginous microorganisms are named as single cell oils (SCO). Most of these oil accumulating microorganisms are species of yeast and fungi. The comprehensive nuclear ribosomal deoxyribonucleic acid (DNA) molecular phylogeny analysis reported that the order *Mortierellales* contains nearly 100 described species and the *Mortierellaceae* family contains about 13 genera. *M. alpina* is one of the main single cell oil producing species/arachidonic acid producing at commercial scale under *Mortierella* genus. ( Coemans 1863; Hibbett et al. 2007; Hoffman et al. 2013; Spatafora et al. 2016; Wagner et al. 2013). Wang et al. (2011) described the *M. alpina* genome scale reconstructed metabolic model for higher production of arachidonic acid at industrial scale. Scientists are in continuous search for the new species/novel strains and trying hard to crack the reconstruction genome code to exploit these species, so that the arachidonic acid production can be simplified and commercialized with an improved protocol ( Shin et al. 1994; Rhie et al. 2002; Rhie and Park 2001; Ha et al. 2004).

Oleaginous fungi especially *Mortierella* species are ubiquitous, saprophytic and belong to zygomycetes class. The polyunsaturated fatty acids (PUFA) production potential makes these fungi unique and significant to oil producing industries. Modern internal transcribed spacer based taxonomical classification ( Kirk 1997; Linnemann 1941; Degawa and Gams 2004; Ariyawansa et al. 2015) categorizes the *Mortierella* genus into seven groups: selenospora and parvispora”, “verticillata-humillis”, “lignicola”, “mutabillis, globulifera and angusta”, “strangulate and wolfii”, “alpina and polycephala”, and “gamsii”.

During our studies on Libyan Mortierellaceous fungi, we have isolated many diverse species. Surprisingly, four species of *Mortierella* we have encountered in Libya have not yet been reported. To our knowledge, this is the first report on these oleaginous fungal species from this country.

## Methods

### Collection of soil samples and isolation of fungi

This study was carried out in December 2017. In total, four different locations *viz.* Marawah, Albayda, Faydiyah and Suluntah located in district Aljabal Al-Akhdar, North-East Libya were chosen as shown in Table 1. In total, a 10 g rhizosphere soil sample from each location was collected in sterilized polybags and transported to the microbiology laboratory of Management and Science University, Shah Alam, Malaysia and stored at 4°C for further processing.

**Table 1.** Description of geographical coordinates collection sites in Al-Jabal Alakdar (Northeast Libya).

The location	Number of samples	Date of collection	Places of collection	Temperatures in months of collection (°C)		Average annual temperature (°C)	Average annual rainfall (mm)	Average annual relative humidity (%)	Geographical distribution (Coordinates)	Altitude (m)
				December (Min-Max)	January (Min-Max)					
Al-Jabal Alakdar (Northeast of Libya)	Sample 1	13.12.2017	Marawah	7.0-15.6	4.9-13.4	16.4	256	68	32°28′59″N 21°24′15″E	464
	Sample 2	16.12.2017	Albayda	6.1-14.5	4.0-11.8	15.3	540	67	32°45′59″N 21°44′30″E	624
	Sample 3	16.12.2017	Faydiyah	5.0-13.5	2.9-11.0	14.2	405	68	32°41′26″N 21°54′27″E	774
	Sample 4	16.12.2017	Suluntah	5.1-13.6	3.1-11.3	14.4	408	69	32°35′25″N 21°42′57″E	754

The fungal isolation was carried out by a conventional serial dilution technique in which 1 g of soil was mixed with 9 mL of sterile distilled water and shaken for 15 min at 25°C; serial dilutions ranging from 10 ^−1^ to 10 ^−4^ were made. An aliquot of 0.1 mL from each dilution was transferred to potato dextrose agar supplemented with 100 μg chloramphenicol/mL antibiotic and incubated at 25°C for 3–7 days.

### Morphological identification

Morphological features of the fungus were observed on potato dextrose agar (PDA) medium after one-point inoculation in 9-cm petri dishes and incubation at 25°C for 5-7 days ( Hyde et al. 2016). The samples were inoculated with the help of a sterilized inoculation needle by center point inoculation on the PDA media containing petri dishes. All methods were performed at the laminar air flow by maintaining all aseptic conditions to avoid any kind of contamination using standard protocol described by Lee et al. (2017). The Petri dishes were sealed by parafilm and incubated for 5-7 days at 25°C in the dark for the growth of novel fungal species. All four distinct isolated fungal species were kept on plastic Petri dishes (9 cm diameter). These plates were observed on daily basis and their morphological characteristics *viz.* colony appearance, pigmentation, growth pattern, colony colour (front and reverse), colony diameters were documented. Individual colonies of fungi that showed varying morphologies were picked up and identified by mycokeys 3.0 version. The morphological features of four fungal isolates were compared with distinguished monographs precisely with II Subgenus: *Mortierella*; 2. Section ALPINA Linnem. Mucorineen-Gatt. *Mortierella*: 35. 1941 monograph ( Gams 1977) to assess the novelty as shown in Table 2.

**Table 2.** Comparison of morphological and cultural characteristics of fungal isolates obtained in this research study with reference, *Mortierella alpina* ^**a**^.

Characteristics	MSU-101 (No. MZ298831)	MSU-201 (No. MZ298832)	MSU-401 (No. MZ298834)	MSU-501 (No. MZ298835)	*Mortierella alpina* ^**a**^ M136 (ATCC 32222; CBS 528.72)
Colony	Rapidly growing at 25°C on PDA, whitish colour; reverse colour of colony light yellowish white and little zonate pattern	Rapidly growing at 25°C on PDA, Slightly cottony at the center with white margin; reverse colour of colony yellowish white with moderately zonate pattern	Rapidly growing at 25°C on PDA, Cottony at the center with white margin; reverse colour of colony slightly yellowish white with irregularly zonate pattern	Rapidly growing at 25°C on PDA, Cottony growth at the center with whitish margin; reverse colour of colony dark yellowish white overlapping in-distinguished zonate pattern	Cobweb to cotton-like White, arachnoid to cottony
Sporangiophores	Moderately branched, 2-3.5 μm wide at tip with variable length, Upto 245 (−370) μm long	Mostly branched, 3-3.5 (−2) μm wide at tip with variable length, Upto 250 (−400) μm long	Mostly branched, 3.3-3.8 (−2) μm wide at tip with variable length, Upto 250 (-390) μm long	Mostly branched, 3.3-3.8 (−2) μm wide at tip with variable length, Upto 250 (−400) μm long	1.5-3.5 μm wide at tip with variable length, 5-8 (−12), Upto 250 (−400) μm long
Sporangia	Globose, multi-spores, 16.5-33.5 × 18-32 μm	Globose, multi-spores, 16-32 × 19-32 μm	Globose, multi-spores, 14-33.5 × 18-32 μm	Globose, multi-spores, 16.5-33.5 × 18-32 μm	Globose, (15−) 20-30 μm
Sporangiospores	Oval, smooth, hyaline 8-15.5 × 5-8.5 μm	Ovoid, smooth, hyaline 7-14.5 × 4.8-8.3 μm	Ovoid, smooth, hyaline 7-14.5 × 5-8.5 μm	Ovoid, smooth, hyaline 7.5-15.5 × 5-8.5 μm	Ovoid, smooth, hyaline 5-11 × 5-9.5 μm
Chlamydospores Zygospores	Present Not observed	Present Not observed	Present Not observed	Present Not observed	Present Globose to sub-globose, (42−)55(−80) × (40−)52(−70) μm

^a^ Source of reference: Gams W. (1977) and Nagy et al. (2011).

Direct microscopic identification was performed by using distilled water (wet mount technique) in which, a clean glass slide was labelled in the middle portion by marker and a drop of sterilized distilled water was put in on the marked middle portion, aerial spores and vegetative hyphae of the fungal isolate taken with the help of sterilized inoculation needle and distributed evenly within the water drop. Subsequently the glass coverslip was carefully added on the preparation in such a way that there was no air bubble formed. Same procedure was applied with lactophenol solution for identification of distinguished structures and prepared slides were examined under a light microscope at 40× magnification (Model: SZX16 Olympus, Japan). The sporangiophore, sporangium and sporangiospores, shape and size, developmental pattern, mature and immature sporangiospores, intercalary chlamydospore were measured and documented ( White et al. 1990). Pure cultures of four fungal isolates were preserved and maintained (Fully grown barcoded fungal cultures after 5 days incubation at 25°C) in PDA slant tubes and stored in 20% glycerol at –80°C in a cold chamber of the university microbiology laboratory. Later, all four cultures were barcoded as MSU-101, MSU-201, MSU-401 and MSU-501 and deposited at MSU Culture Collection Center, Management and Science University, Shah Alam, Selangor, Malaysia.

### Genomic DNA extraction and sequence alignment

Total genomic DNA (gDNA) was extracted according to the standardized protocol ( Tamura et al. 2013). ITS and rDNA conserved regions were amplified using ITS4 (5′-GGAAGTAAAAGTCGTAACAAGG-3′) and ITS1 (5′-TCCTCCGCTTATTGATATGC-3′)

Total genomic DNA (gDNA) was extracted directly from the mycelia of fungal isolates, using Genomic DNA reparation Kit (KIT-1200-50: Fungal DNA Barcoding Kit, Apical Scientific Sdn Bhd Malaysia, following the manufacturer’s instructions). Step by step protocol of gDNA isolation includes 1. 500 μL of Fungal Lysis Buffer added into 1.5 mL micro-centrifuge tube that contains the 1 cm agar cube of pure fungal culture. 2. 3 μL of Proteinase K solution added. Vortex to mix and spin down briefly. 3. The tubes were incubated at 56° C for overnight and centrifuged the lysate at 14,000 to 16,000×g for 10 minutes. 4. Transferred ~500 μL of supernatant to a new 1.5 mL micro-centrifuge tube, which contains 500 μL of isopropanol. The tube was inverted several times to mix gently. 6. Centrifuged at 14,000 to 16,000×g for 10 min and the supernatant was discarded. 7. 1 mL of 70% ethanol was added, centrifuged again at 14,000 to 16,000×g for 5 min and the supernatant was discarded. 8. The pallet was air dried for 3 min, resuspended with 50 μL TE Buffer and incubated at 56°C for < 1 hr. 9. Optical density (OD) was measured reading using spectrophotometer (Thermo Scientific, USA) and the nucleic acid was diluted to 15 to 25ng/μL and 2 μL of diluted nucleic acid was used as DNA template for PCR. 10. PCR mix was prepared according to manufacturer’s instructions and 2 μL of DNA template was added with each 23 μL of PCR mix into 0.2 mL tube or 96-well plate. 11. PCR cycle protocol was run on thermocycler and ~700bp PCR products were checked on 1% agarose gel (First Base NGS KIT, Malaysia) and sequenced by ABI3100 sequencer. 12. After the sequencing results were ready, the reads were trimmed off with quality value (QV) < 20, after that the forward and reverse sequencing results were aligned. 13. The obtained sequences were compared against the earlier submitted NCBI database using the BLAST algorithm ( Kimura 1980) to verify the percentage of identity corresponding to the analysed species ( Table 2). 13. The fungal sequences were aligned using Clustal_X v.2.1 and neighbour joining based phylogenetic tree was constructed using Mega (molecular evolutionary genetic analysis) X software version 16.04.4 (with unity desktop, ANALYZE mode; Tamura et al. 2013) to observe the grouping of obtained novel fungal species sequences ( Kimura 1980; Nagy et al. 2011; Chien et al. 1974).

## Results and discussion

### Morphological confirmation

**Figure 1.**
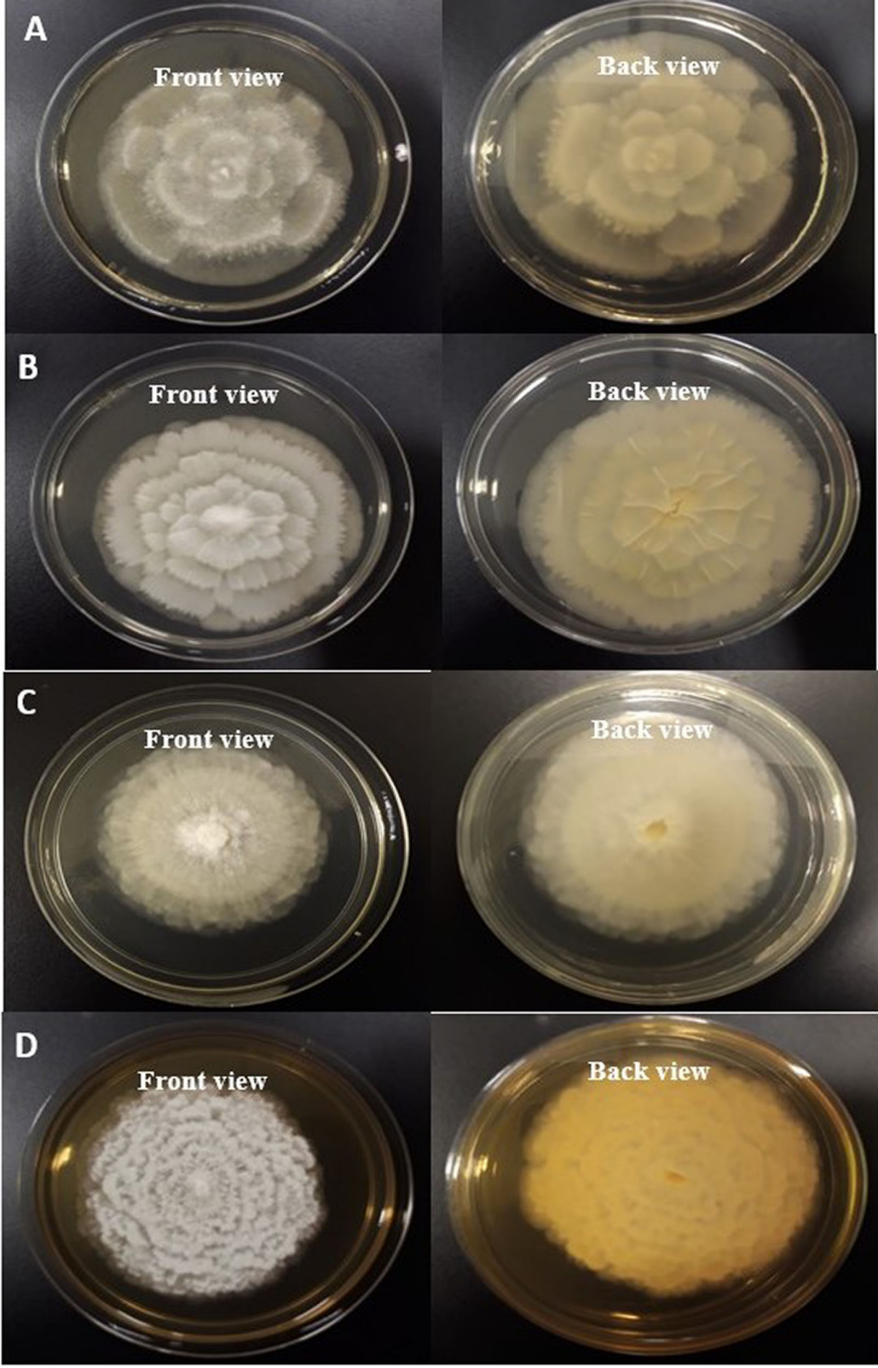
Colonies of fungal isolates on potato dextrose agar (PDA) medium (front view and back view) after 7 days of incubation at 25°C. (A) MSU-101 colonies on PDA front and back view. (B) MSU-201 colonies on PDA front and back view. (C) MSU-401 colonies on PDA front and back view. (D) MSU-501 colonies on PDA front and back view.

**Figure 2.**
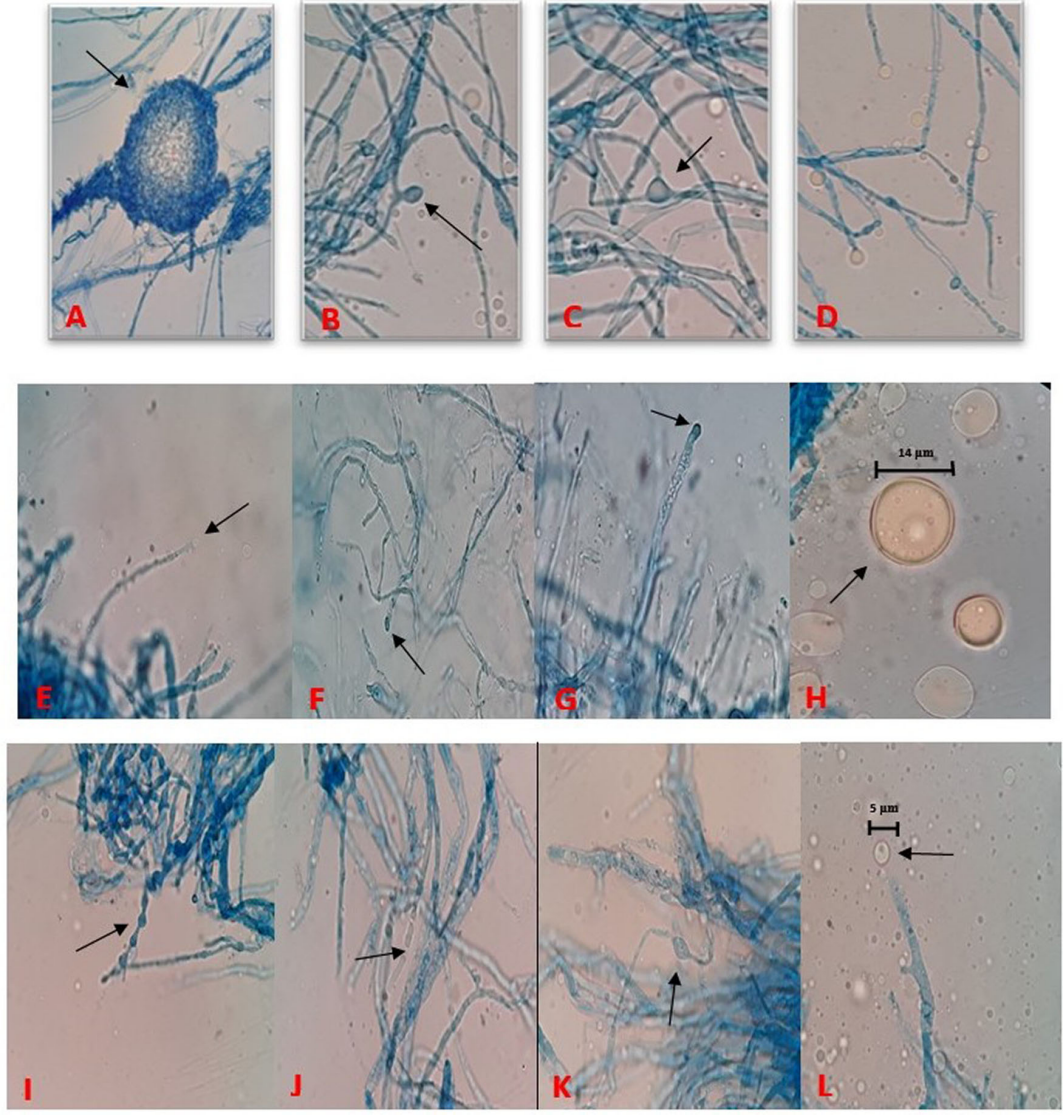
(A) Meiospore of MSU-101 isolate. (B) Immature sporangia from branched sporangiophore of MSU-101. (C) Intercalary chlamydospore of MSU-101 isolate. (D) Hyaline and ovoid sporangia, MSU-201 isolate. (E) Developing sporangia on single sporangiophore, MSU-201 isolate. (F) Immature young sporangia on highly branched sporangiophore, MSU-201. (G) Immature young sporangia on highly branched sporangiophore, MSU-401. (H) Mature globose sporangium containing sporangiospores, MSU-401. (I) Terminal chlamydospores with papillate ornamentation and hyphal segment remaining at the distal end, MSU-501. (J) Net of hypha with branching and septation, MSU-501. (K) Net of hypha with branching and chlamydospore, MSU-501. (L) Developing sporangium at tip on sporangiophore, MSU-501.

On the basis of morphological and cultural characteristics, the fungal isolates were confirmed and belong to *Mortierella* genus. Colonies of oleaginous fungal isolates after seven days of incubation at 25°C on PDA, were sporulating, fast growing, producing a concentric pattern, had flower-shaped radial growth, and were yellowish to whitish in color as depicted in Figure 1 ( Eltariki, Tiwari, & Alhoot, 2021). The detailed descriptions of morphological characteristics such as sporangiophores, sporangium, sporangiospores with reference *M. alpina* (ATCC 32222; CBS 528.72) isolate are given in Table 3 and Figure 2. Distinguishing prominent features between four fungal isolates (Barcoded as MSU-101, MSU-201, MSU-401 and MSU-501) were growth pattern, margin and colour of the colony on PDA medium in front and back side as shown in Figure 1, which requires further investigation. Thus, these four novel isolates were examined for molecular characterization and genetic diversity.

**Table 3.** GenBank accession numbers used for the phylogenetic analyses in the present study.

Location	Type of sample	Barcode of isolate	Accession number	Percentage (%) of similarity by Clustal_X
Austria	Environmental sample	Uncultured *Mortierella* Clone IIS1-5	EU517021	100
**Marawah, North-East Libya**	**Soil sample**	** *Mortierella alpina* Strain MSU-101**	**MZ298831**	**100**
China	Soil sample	*Mortierella alpina* Strain QLF48	FJ025186	100
Aragon, Spain	Calcareous soil and Tuber Melanosporum ectomycorrhizal in the Mediterranean Zone	*Mortierella alpina* isolate MM3	KX343169	99.83
**Suluntah, North-East Libya**	**Soil sample**	** *Mortierella alpina* strain MSU-401**	**MZ298834**	**100**
Tongshan: New District, Xuzhou, Jiangsu, China	(Endophytic fungi) Seed sample	*Mortierella alpina* strain xsd08339	EU918703	99.83
Tianshui Lanzhou, Gansu, China	Endophytic fungi from the rhizosphere soils and roots of *Lycium barbarum* L.	*Mortierella alpina* strain GFRS11	MT447479	99.67
Tianshui Lanzhou, Gansu,China	Endophytic fungi from the rhizosphere soils and roots of *Lycium barbarum* L.	*Mortierella alpina* strain QLF60	FJ025143	99.67
Tianshui Lanzhou, Gansu, China	Endophytic fungi from the rhizosphere soils and roots of *Lycium barbarum* L.	*Mortierella alpina* strain QLF70	FJ025182	99.83
Mainz, Germany	Soil Sample	*Mortierella alpina* isolate A03ID2	KJ469805	98.85
Wageningen, Netherlands	Lyophilized spore material from the CBS-KNAW Fungal Biodiversity Centre in Utrecht, the Netherlands.	*Mortierella alpina* isolate d27	GQ922556	98.85
Lanzhou, Gansu, China	Endophytic fungi from the rhizosphere soils and roots of *Lycium barbarum* L.	*Mortierella alpina* strain QLF27	FJ025187	99.5
Qingdao, China	Soil Sample from Antarctica	*Mortierella* sp. strain HSX2#-13	MT367225	98.84
Asahikawa, Hokkaido, Japan	Samples from Walker glacier, Canadian High Arctic	*Mortierella alpina* GR8-3-20-1	LC515164	99.5
Larisa, Greece	Microbial community from rhizosphere soil sample	Uncultured zygomycete clone 1B6	FN689671	100
Lanzhou, Gansu, China	Endophytic fungi from the rhizosphere soils and roots of *Lycium barbarum* L.	*Mortierellales* sp. strain GFRS01	MT447469	99.84
Turin, ITALY	Environmental sample	Uncultured fungus clone 62	FN391358	99.84
**Albayda, North-East Libya**	**Soil Sample**	** *Mortierella alpina* strain MSU-501**	**MZ298835**	**100**
**Faydiyah, North-East Libya**	**Soil Sample**	** *Mortierella alpina* strain MSU-201**	**MZ298832**	**100**
Lanzhou, Gansu, China	Soil Sample from alpine grassland in eastern Qilian mountains	Mortierellales sp. QLF86	FJ025158	100
Larisa, Greece	Microbial community from rhizosphere soil sample	Uncultured Mortierellales clone 2B12	FN689675	100
Haidian district, Beijing, China	Soil Sample	*Mortierella alpina* strain XY01520	MT521795	100
Berlin, Germany	Fine airborne particles/spores, environmental Sample	*Mortierella alpina* isolate DSM100289_DF19_RLCS11	MT453274	100
Gronostajowa 7, Krakow, Malopolska, Poland	Rhizosphere Soil Sample Symbiotic microbes of Saxifraga stellaris sp. alpigena from the copper creek of the Schwarzwand (Austrian Alps)	*Mortierella* sp. isolate MMS	MF565377	100
Sevilla, Spain	Environmental sample	Uncultured *Mortierella* clone IB2_K7	FN812729	100
Viale Mattioli, Italy	Environmental sample	Uncultured fungus clone iO03_P_2_B12	FN397316	100
Anning District, Lanzhou, Gansu, China	Soil Sample from alpine grassland in eastern Qilian mountains	*Mortierellales* sp. QLF84	FJ025170	100
Viale Mattioli, Italy	Environmental sample	Uncultured fungus clone iE12_P_2_D7	FN397313	99.84
Innsbruck, Tyrol, Austria	Environmental sample	Uncultured *Mortierellaceae* clone IIS4-1	EU517031	100
Viale Mattioli, Italy	Environmental sample	Uncultured fungus clone 58	FN391354	99.84
Anning District, Lanzhou, Gansu, China	Soil Sample from alpine grassland in eastern Qilian mountains	*Mortierellales* sp. QLF15	FJ025162	100
Viale Mattioli, Italy	Environmental sample	Uncultured fungus clone 50	FN397151	100
Av. Monta nana, Zaragoza, Spain	Diversity of fungi isolated of calcareous Soil Sample and *Tuber melanosporum*	*Mortierella alpina* isolate 20PDA-D30	KX343151	99.84
Halle/Saale, Germany	Environmental sample	Uncultured Mortierella clone 09S50C12 (MOTU44)	HG936566	100

The newly submitted novel *Mortierella alpina* strains ITS sequences in GenBank are indicated in bold.

### Molecular characterization and genetic diversity

**Figure 3.**
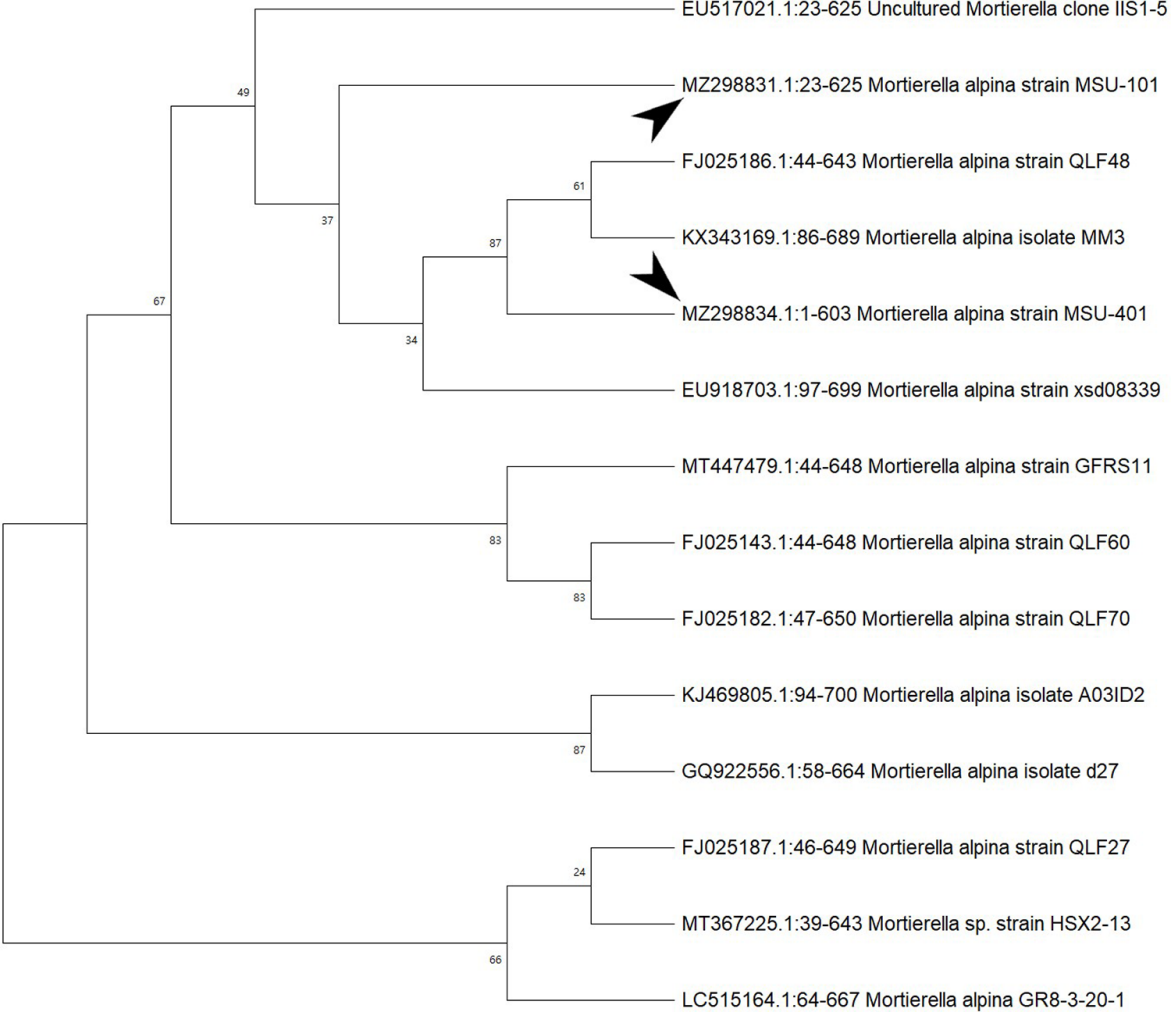
Neighbour joining method based phylogenetic tree from internal transcribed spacer conserved sequences of isolates MSU-101 and MSU-401. Bootstrap support values are indicated at the nodes.

**Figure 4.**
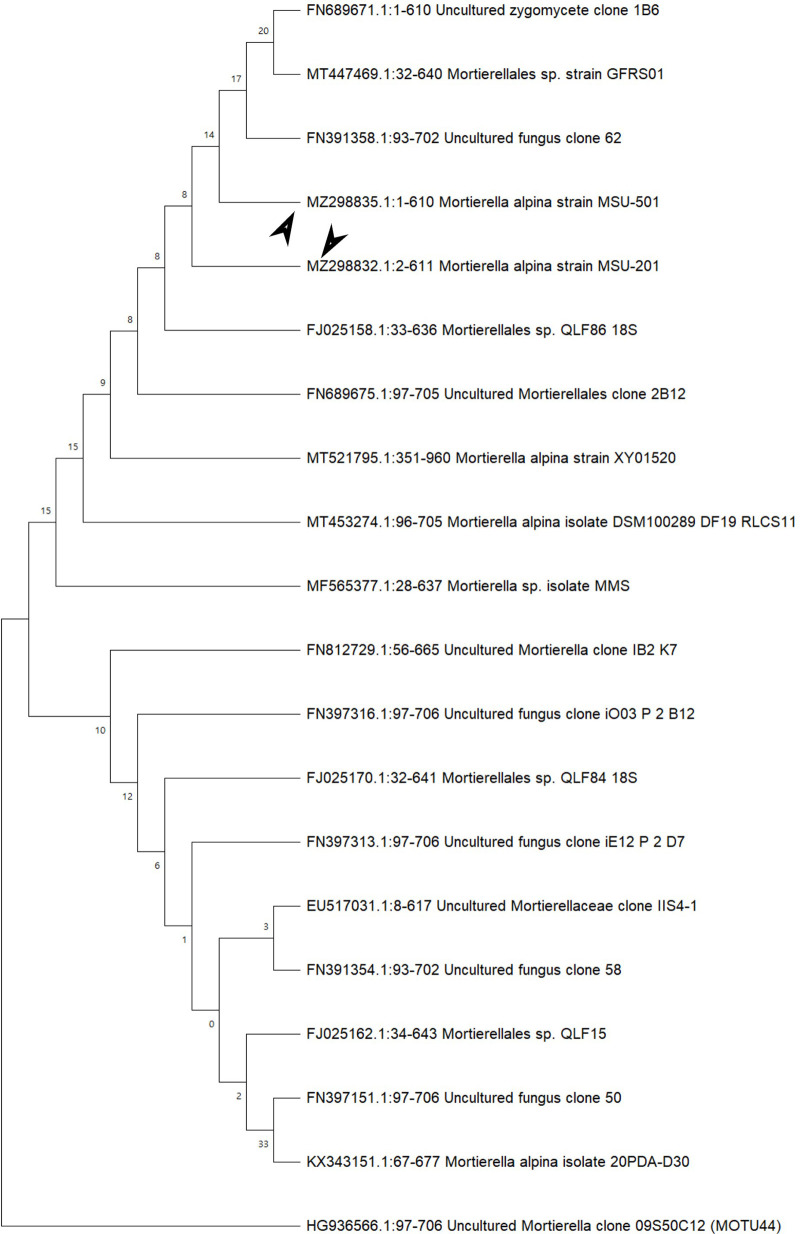
Neighbour joining method based phylogenetic tree from internal transcribed spacer conserved sequences of isolates MSU-201 and MSU-501. Bootstrap support values are indicated at the nodes.

In the ITS sequences analysis based on BLASTn (Basic Local Alignment Search Tool for nucleotides), MSU-101, MSU-201, MSU-401 and MSU-501 isolates were fall within the order *Mortierellales* as depicted in Figures 3 and 4, which matches with morphological identification of isolates as described above. These four fungal isolates (barcoded as MSU-101, MSU-201, MSU-401 and MSU-501) were compared and aligned with earlier submitted closely related species sequences by multiple sequence alignment (FASTA format) with software Clustal_X v.2.1. The phylogenetic tree constructed by neighbour joining mode with 1000 bootstrap values, showed that four oleaginous fungal isolates were 100% similar with earlier *M. alpina* genomes sequences submitted in GenBank NCBI (closest matching GenBank accession numbers were: EU918703; KX343169; FJ025186; FN689671; FN391358; FJ025158) as shown in Table 2 and Figures 3 and 4. Thus, these isolates were identified as *M. alpina* species. The ITS sequences of these fungal isolates were deposited in GenBank with accession number of MZ298831:MZ298835.

The present study added on the *Mortierella alpina* fungal strains reference collections and describes the diversity of these strains with known strains to date as shown in Figures 1 and 2. These novel *Mortierella* isolates add on to a large contribution of fungal diversity collections all over the world but still there is a plenty of room for more comprehensive *M. alpina* collections from Libya and this is the limitation of the present study. Thus, further research work needs to be carried out in future so that the hidden *Mortierella* fungal diversity and their SCO production potential can be harnessed.

Chen and Ho (2008) reported the significance of internal transcribed spacer region (18S-28S ribosomal gene) for the genetic characterization and these strains and found that the 5.8 rDNA regions of *M. alpina* isolates were conserved except some identified polymorphic sites. Furthermore, the interpretation was made that the variability is present in ITS1 and ITS2 regions, as there was no polymorphic site in the 5.8 rDNA region. Thus, it was evident that the ITS region could be used to confidently discriminate between *M. alpina* and other closely related species. These researchers also highlighted that NJ (Neighbour-Joining phylogenetic tree) tree analysis provides precise genetic diversity between *M. alpina* strains to come out with significant interpretation and conclusion.

Many species of *Mortierella* are potentialistic producers of C18 and C20 PUFAs (polyunsaturated fatty acids) such as γ-linolenic acid and arachidonic acid. *M. alpina* species is quite famous for the production of single cell oils as describes and reported by multiple scientist’s time by time ( Chien et al. 1974; Huang et al. 2013; Tamayo-Velez and Osorio 2018; Osorio and Habte 2014; Ellegaard et al. 2013; Lee et al. 2015; Nguyen and Lee 2016; Hwang et al. 2005; Shin et al. 2005; Tiwari and Razip 2020; Tiwari and Ganesen 2020; Maitig et al. 2028; Khan et al. 2018; Asdren and Faizal 2018; Yu et al. 2019; Alhoot et al. 2019; Tiwari et al. 2018, 2019a, b).

Research scientists are working to remodel these novel strains so that the SCO production can be enhanced at industrial scale. Shimizu and Sakuradani (2009) reported *M. alpina* 1S-4 strain by extensive screening, for the large-scale production of variety of PUFAs. This isolate not only had the potential for SCO production but also had several advantages to work as a model for lipogenesis studies. Thus, we can anticipate from earlier published data that the isolates reported from present study can be useful for bioprospecting in terms of single cell oil production. However, the oil production potential of these oleaginous fungal isolates is under investigation and our research group is presently working in this direction to assess the SCO potential of these diverse isolates obtained from Libyan soil.

## Conclusion

In the present study, four oleaginous fungal isolates barcoded as MSU-101, MSU-201, MSU-401 and MSU-501 were identified and confirmed by morphological and molecular analysis. These fungal isolates had shown highest similarity with *Mortierella alpina* species and can be potential single cell oil producers, further research work is in progress for assessment and exploitation of these isolates in terms of oil production.

## Data availability

NCBI GenBank: Accession numbers MZ298831 to MZ298835.

https://www.ncbi.nlm.nih.gov/nuccore/?term=MZ298831:MZ298835[accn].

Zenodo: Molecular characterization and genetic diversity of four undescribed novel oleaginous Mortierella alpina strains from Libya. https://doi.org/10.5281/zenodo.5239888 ( Eltariki, Tiwari, & Alhoot, 2021).

This project contains the following underlying data:

- Developing sporangia on single sporangiophore, MSU-201 isolate.jpg
- Developing sporangium at tip on sporangiophore, MSU-501.jpg
- Hyaline and ovoid sporangia, MSU-201 isolate.jpg
- Immature sporangia from branched sporangiophore of MSU-101.jpg
- Immature young sporangia on highly branched sporangiophore, MSU-201.jpg
- Immature young sporangia on highly branched sporangiophore, MSU-401.jpg
- Intercalary chlamydospore of MSU-101 isolate.jpg
- Meispore of MSU-101 isolate.jpg
- Mortierell alpina (4 strains) gel image.jpg
- Mortierella alpina novel strain-MSU-201_Front view.jpg
- Mortierella alpina novel strain_MSU-101.jpg
- Mortierella alpina novel strain_MSU-101_Back view.jpg
- Mortierella alpina novel strain_MSU-201_Back view.jpg
- Mortierella alpina novel strain_MSU-401_Back view.jpg
- Mortierella alpina novel strain_MSU-401_Front view.jpgMortierella alpina novel strain_MSU-501_Back view.jpg
- Mortierella alpina novel strain_MSU-501_Front view.jpg
- Net of hypha with branching and chlamydospore, MSU-501.jpg
- Net of hypha with branching and septation, MSU-501.jpg
- Phylogenetic tree MSU-101 and MSU-401.jpg
- Phylogenetic tree_MSU-201 and MSU-501.jpg
- Terminal chlamydospores with papillate ornamentation and hyphal segment remaining at the distal end, MSU-501.jpg
- Terminal chlamydospores.jpg

Data are available under the terms of the Creative Commons Attribution 4.0 International license (CC-BY 4.0).
