# Supplementary material for: Molecular characterization and genetic diversity of four undescribed novel oleaginous Mortierella alpina strains from Libya
Source: F1000Res. 2021 Sep 7;10:895. [Version 1] doi: 10.12688/f1000research.70644.1 (PMC8564746; doi:10.12688/f1000research.70644.1)
Supplement: Peer review history

# Peer review: Reviewer response for version 1

**DOI:** 10.5256/f1000research.74246.r93646

**Published:** 2021-11-02

**Authors:** Abhijeet Singh

**Affiliations:** 1 Department of Biosciences, Manipal University Jaipur, Jaipur, India

The research study addresses and discusses the novel strains isolation, identification and genetic diversity assessment by standard protocols. The conserved internal transcribed spacer regions amplified and probably gives idea about the specific region in internal transcribed spacer for loci in terms of bioprospecting and exploitation of these novel strains for single soil production. The methodology discusses the isolation, identification and molecular characterization of *M. alpina* strains from Al-Jabal Alakdar (Northeast of Libya). The novel strains sequences submitted in NCBI GenBank with specific barcode (barcoded as MSU-101, MSU-201, MSU-401 and MSU-501) (accession no. MZ298831: MZ298835) and cultures preserved at Culture Collection Center of University. Thus, the research work is accepted and recommended for indexing.

Is the work clearly and accurately presented and does it cite the current literature?

Yes

If applicable, is the statistical analysis and its interpretation appropriate?

I cannot comment. A qualified statistician is required.

Are all the source data underlying the results available to ensure full reproducibility?

Yes

Is the study design appropriate and is the work technically sound?

Yes

Are the conclusions drawn adequately supported by the results?

Yes

Are sufficient details of methods and analysis provided to allow replication by others?

Yes

Reviewer Expertise:

Molecular Biology, Bionanotechnology

I confirm that I have read this submission and believe that I have an appropriate level of expertise to confirm that it is of an acceptable scientific standard.

# Peer review: Reviewer response for version 1

**DOI:** 10.5256/f1000research.74246.r93650

**Published:** 2021-11-02

**Authors:** Digpal Singh Gour

**Affiliations:** 1 Department of Fish and Wildlife Sciences, University of Idaho, Moscow, ID, USA; 2 Genomic Resource Core, Institute for Bioinformatics & Evolutionary Studies, University of Idaho, Moscow, ID, USA

The research study documented four novel *M. alpina* strains to produce single cell oil and describes the isolation, identification, and molecular characterization of *M. alpina* strains from al-Jabal al-Akhḍar (Northeast of Libya) by standard protocols. The conserved internal transcribed spacer regions amplified, and assessment of genetic diversity carried out in this research work.

 The novel strains sequences submitted in NCBI GenBank with specific barcodes (barcoded as MSU-101, MSU-201, MSU-401, and MSU-501) (accession no. MZ298831: MZ298835) were given.

 However, further research work in connection with single cell production can be done and set a benchmark for bioprospecting in the future. I hope in future the work will be carried out in this direction. As far as the present research study is concerned, the novel strains are described, organized, and documented, thus accepted for indexing.

Is the work clearly and accurately presented and does it cite the current literature?

Yes

If applicable, is the statistical analysis and its interpretation appropriate?

Not applicable

Are all the source data underlying the results available to ensure full reproducibility?

Yes

Is the study design appropriate and is the work technically sound?

Yes

Are the conclusions drawn adequately supported by the results?

Yes

Are sufficient details of methods and analysis provided to allow replication by others?

Yes

Reviewer Expertise:

Genetics & Genomics

I confirm that I have read this submission and believe that I have an appropriate level of expertise to confirm that it is of an acceptable scientific standard.

# Peer review: Reviewer response for version 1

**DOI:** 10.5256/f1000research.74246.r93648

**Published:** 2021-09-17

**Authors:** Kavindra Kumar Kesari

**Affiliations:** 1 Department of Applied Physics, School of Science, Aalto University, Espoo, Finland

**Comments**:

 Justification on the research work shown is manuscript is profound and significant since the research manuscript describes valuable information on *Mortierella alpina* novel strains isolated from Libya.

 Stepwise flowsheet and methodology discuss the isolation, identification and molecular characterization of *M. alpina* strains from Al-Jabal Alakdar (Northeast of Libya).

*Mortierella alpina* strains are well known and documented for the potential of single cell oil production (SCO) at large scale. So, the conserved internal transcribed spacer regions amplification and assessment of genetic diversity carried out in this research work will give insight on future standard protocols and further work in this field for exploitation and production of arachidonic acid/poly-unsaturated fatty acids production. The novel strains sequences submitted in NCBI GenBank with specific barcode (barcoded as MSU-101, MSU-201, MSU-401 and MSU-501) (accession no. MZ298831: MZ298835) and cultures preserved at Culture Collection Center of University.

 Further research work in connection with single cell production can be done and set a benchmark for bioprospecting in future. The work was carried out by a PhD candidate and will facilitate the career growth for the candidate for future opportunities and prospects.

Is the work clearly and accurately presented and does it cite the current literature?

Yes

If applicable, is the statistical analysis and its interpretation appropriate?

Yes

Are all the source data underlying the results available to ensure full reproducibility?

Yes

Is the study design appropriate and is the work technically sound?

Yes

Are the conclusions drawn adequately supported by the results?

Yes

Are sufficient details of methods and analysis provided to allow replication by others?

Yes

Reviewer Expertise:

Cell and cancer biology; plant biotechnology; industrial microbiology

I confirm that I have read this submission and believe that I have an appropriate level of expertise to confirm that it is of an acceptable scientific standard.
